# Mechanochemical synthesis of Si/Cu_3_Si-based composite as negative electrode materials for lithium ion battery

**DOI:** 10.1038/s41598-018-30703-3

**Published:** 2018-08-23

**Authors:** Shang-Chieh Hou, Tsan-Yao Chen, Yu-Hsien Wu, Hung-Yuan Chen, Xin-Dian Lin, Yu-Qi Chen, Jow-Lay Huang, Chia-Chin Chang

**Affiliations:** 1 Department of Materials Science and Engineering, National Cheng Kung University, Tainan, 70101 Taiwan; 2 Department of Engineering and System Science, National Tsing Hua University, Hsinchu, 30013 Taiwan; 3 Department of Greenergy, National University of Tainan, Tainan, 70005 Taiwan; 4 Center for Micro/Nano Science and Technology, National Cheng Kung University, Tainan, 70101 Taiwan; 5 Hierarchical Green-Energy Materials (Hi-GEM) Research Center, National Cheng Kung University, Tainan, 70101 Taiwan

## Abstract

Mechanochemical synthesis of Si/Cu_3_Si-based composite as negative electrode materials for lithium ion battery is investigated. Results indicate that CuO is decomposed and alloyed with Si forming amorphous Cu-Si solid solution due to high energy impacting during high energy mechanical milling (HEMM). Upon carbonization at 800 °C, heating energy induces Cu_3_Si to crystallize in nanocrystalline/amorphous Si-rich matrix enhancing composite rigidity and conductivity. In addition, residual carbon formed on outside surface of composite powder as a buff space further alleviates volume change upon lithiation/delithiation. Thus, coin cell made of C-coated Si/Cu_3_Si-based composite as negative electrode (active materials loading, 2.3 mg cm^−2^) conducted at 100 mA g^−1^ performs the initial charge capacity of 1812 mAh g^−1^ (4.08 mAh cm^−2^) columbic efficiency of 83.7% and retained charge capacity of 1470 mAh g^−1^ (3.31 mAh cm^−2^) at the end of the 100^th^ cycle, opening a promised window as negative electrode materials for lithium ion batteries.

## Introduction

Rechargeable lithium-ion batteries (LIBs) has been attracted much attention on energy storage due to its high energy density and long cycle life for increasing demand of portable electric devices, electric vehicles (EV), hybrid electric vehicles (HEV), plug-in hybrid electric vehicles (PHEV) and grid energy storage system^1–3^. However, traditional graphite negative material is limited by its theoretical specific capacity of 372 mAh g^−1^. Thus, a lot of effort are paid to develop next generation materials for negative electrode for LIBs. Silicon is considered to be next generation anode material in lithium ion battery due to its high theoretical specific capacity of 4200 mAh g^−1^ ^4^, low discharge voltage (~0.4 V versus Li^+^/Li), highly abundant resource and low toxicity. However, its huge volume change (>400%) during the lithiation/delithiation results in silicon crumbing and pulverizing, leading to loss of integration of the anode^5^. As a result, silicon negative electrode suffers from more irreversible loss, rapid fading of capacity and short cycle life.

The strategies on improving silicon-based anode materials have been conducted including: (i) silicon nanostructure^6–8^ such as nanoparticles, nanowires, nanotubes, and porous silicon, (ii) silicon composite such as silicon/active^9^ and silicon/inactive composites^10^, (iii) novel silicon architecture such as core/shell^11^, core/hollow/shell^12^. (iv) electrolyte additives^13^ and (v) synthesis of new binders^14^.

Silicon-based composites (silicon/active and silicon/inactive) have been widely studied as buffer space, strength and conductivity enhancement to accommodate volume change upon alloying/dealloying and improve intrinsic low conductivity of silicon. Recently, several works have been published utilizing Cu/copper silicide in silicon-based composites as negative electrode materials in LIBs due to its better conductivity and rigidity compared with silicon^15–21^. As compared to sophisticated synthesis, mechanochemical reaction induced by high energy mechanical milling (HEMM) is known as a robust method conventionally employed in mass-production. HEMM reduce powder size, induce crystallite amorphizing and enable alloying reaction under non-equilibrium driven by high mechanical energy^22^. In our previous work, submicro-sized Si aggregate of nanocrystalline/amorphous phases prepared by combining HEMM with wet milling treatments improve Si negative electrode performance^23^. In this work, Cu_3_Si compound is further synthesized by mechanochemical reaction between CuO and Si upon HEMM. Resulted Cu_3_Si is embedded and dispersed into nanocrystalline/amorphous Si-rich matrix to fabricate Si/Cu_3_Si-based composite. Following, carbonization using glucose as carbon source on composite enhances Cu_3_Si nanocrystallite regardless of nanocrystalline/amorphous Si-rich matrix. Furthermore, residual carbon on composite surface accommodates volume expansion to further improve cycling stability of electrode. Conveniently, both mechanochemical reaction and carbonization are robustly conducted under solid-state condition.

## Results

**Figure 1:**
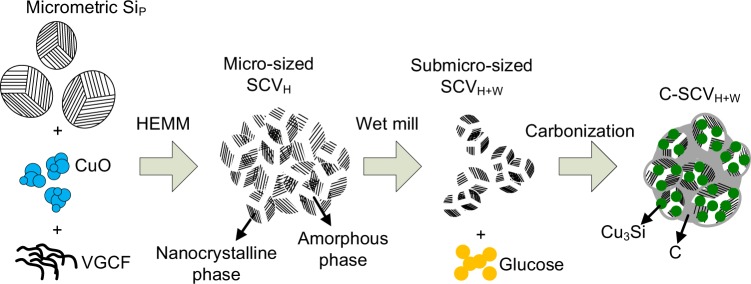
Schematic illustration of the synthesis for Si/Cu_3_Si-based composite powder.

Figure 1 illustrates the synthesis for Si/Cu_3_Si-based composite powder. First, Mixture of micrometric Si_P_, CuO and VGCF was high energy mechanically milled to reduce particle size, which induces amorphizing to form micro-sized SCV_H_ aggregate (nanocrystalline/amorphous phases)^23^. Meanwhile, high energy impacting supposedly leads CuO to decomposing and alloying with Si to form amorphous Cu-Si solid solution. Then, SCV_H_ was wet milled in ethanol to get submicro-sized SCV_H+W_ aggregate. Finally, carbonization using glucose as carbon source at 800 °C, heating energy enabled Cu_3_Si to crystallize from Si-Cu solid solution, resulting in Cu_3_Si distributing into nanocrystalline/amorphous Si-rich matrix for C-SCV_H+W_ aggregate. Besides, residual carbon was covered on surface of C-SCV_H+W_ as buffer layer.

### Powder morphology and TGA analyses

**Figure 2:**
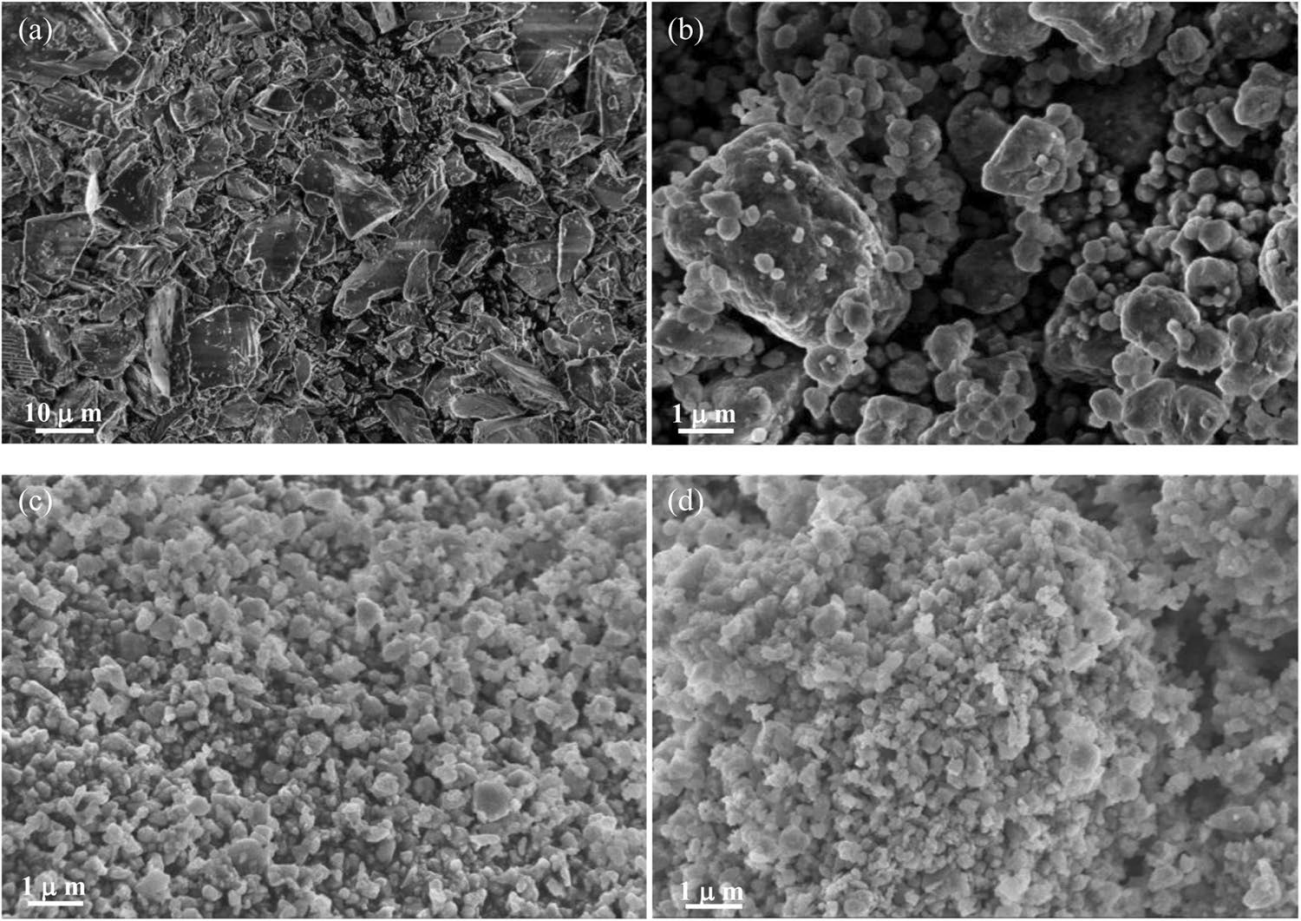
SEM micrographs of the (**a**) Si_P_ , (**b**) SCV_H_, (**c**) SCV_H+W_, and (**d**) C-SCV_H+W_, respectively.

**Figure 3:**
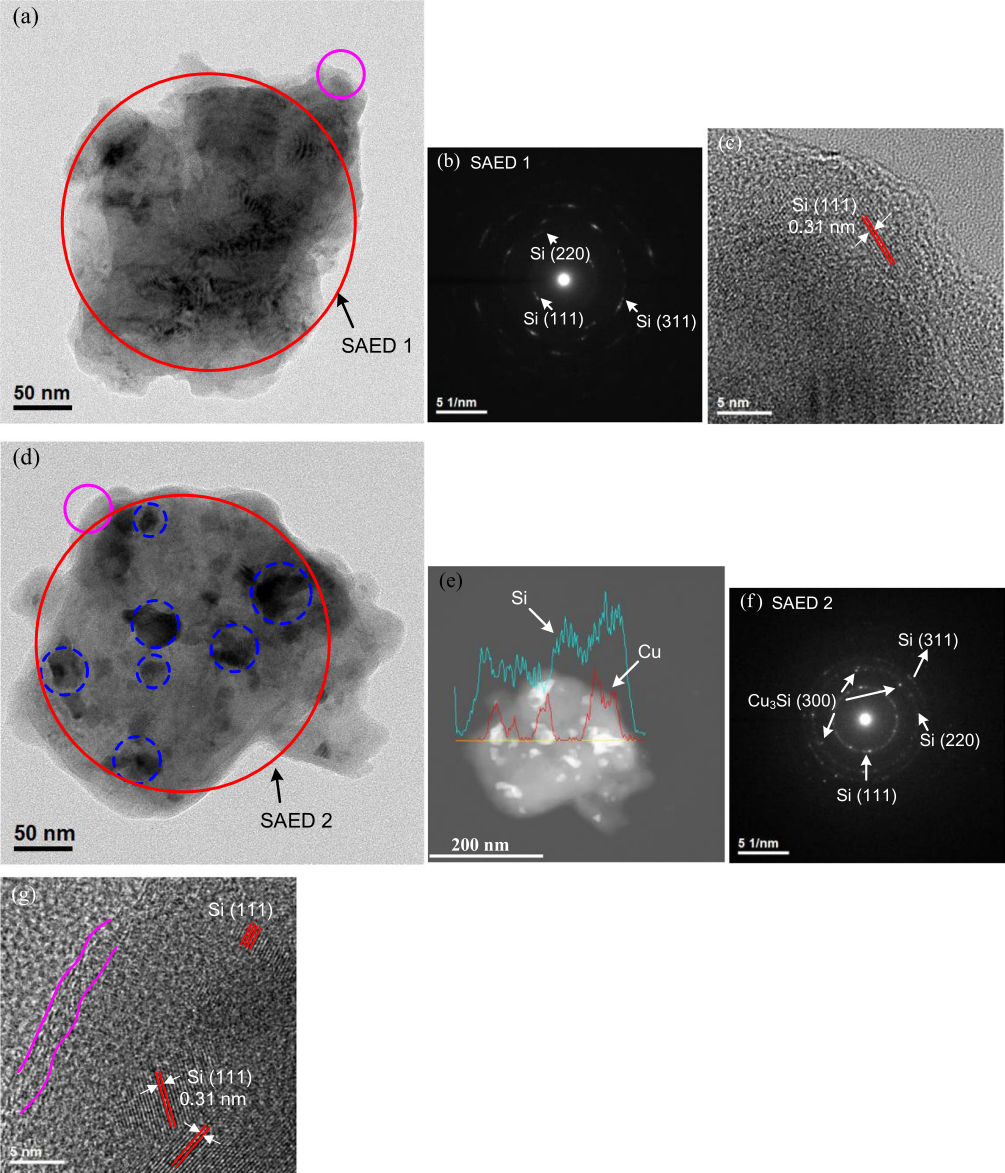
(**a**) TEM image of SCV_H+W_ aggregate, (**b**) SAED 1 pattern, (**c**) HRTEM image referred to pink cycle in (**a)**, (**d**) TEM image of C-SCV_H+W_ aggregate, (**e**) corresponding high-angle annular dark-field image of (**d)**, (**f**) SAED 2 pattern and (**g**) HRTEM image referred to pink cycle in (**d**).

Figure 2 shows the SEM images of Si_P_, SCV_H_, SCV_H+W_ and C-SCV_H+W_. Micrometric Si_P_ presents in irregular flake-like shape (Fig. 2(a)). Upon HEMM, Si_P_, CuO and VGCF are repeatedly fractured and welded to form micro-sized SCV_H_ aggregate constructed by nano-sized primary particles (Fig. 2(b)). SCV_H_ is further reduced to submicro-sized SCV_H+W_ after wet milling in ethanol (Fig. 2(c)). After pyrolysis, C-SCV_H+W_ seems to be slightly adhered via residual carbon due to carbonization of glucose on surface (Fig. 2(d)). Supplementary Fig. S1 presents the SEM-EDS spectrum, revealing that C-SCV_H+W_ is consisted of 75.0 wt.% Si, 10.1 wt.% Cu, 5.3 wt.% C, and 9.6 wt.% O. The detected O could be ascribed to the amorphous SiO_2_ layer on the surface of Si/Cu_3_Si-based composite particles. More detailed observation using transmission electron microscope (TEM) is shown in Fig. 3(a–c) for SCV_H+W_ and Fig. 3(d–g) for C-SCV_H+W_, respectively. Figure 3(a) and (b) present typical submicro-sized SCV_H+W_ and its selected-area electron diffraction (SAED) pattern, SAED 1 corresponding to (111), (220) and (331) of Si without existing others crystalline phases. Figure 3(c) shows the high-resolution transmission electron microscopy (HRTEM) image indexed as pink circle in Fig. 3(a), demonstrating that SCV_H+W_ is constructed by amorphous laminar welds surrounding the nanocrystalline domain of 0.31 nm d-spacing, corresponding to the (111) planes of Si. In contrast, after carbonization, C-SCV_H+W_ presents darker spots that are embedded and dispersed in Si-rich matrix as blue dash line circle indexed in Fig. 3(d). High-angle annular dark-field imaging and line scan of energy dispersive X-ray spectrometry (EDS) reveal that spot areas are highly concentrated by Cu element, suggesting the exhibition of Cu_3_Si as shown in Fig. 3(e). Besides the Si pattern, SAED 2 further confirms the electron diffraction pattern for Cu_3_Si, corresponding to (300) plane as shown in Fig. 3(f). It is consistent with observation of XRD pattern. Figure 3(g) shows the HRTEM image as pink circle indexed in Fig. 3(d), indicating the outside surface of C-SCV_H+W_ is covered by residual carbon originated from glucose carbonization and the inside core of C-SCV_H+W_ is constructed by amorphous/nanocrystalline phases. Therefore, the construction of C-SCV_H+W_ composite could be depicted as the aggregate that nanocrystalline Cu_3_Si disperses in nanocrystalline/amorphous Si-rich matrix. In addition, among these aggregates are associated via residual carbon. Thermogravimetric analysis (TGA) under air atmosphere for C-SCV_H+W_ is shown in Supplementary Fig. S2. The weight of C-SCV_H+W_ is drastically decreased with temperature from 380 °C to 550 °C, which could be ascribe to the weight loss (~4.7 wt%) of residual carbon. After 550 °C, weight of C-SCV_H+W_ is increased with temperature due to Si oxidation.

### Powder crystalline, XPS and ATR-FTIR analyses

**Figure 4:**
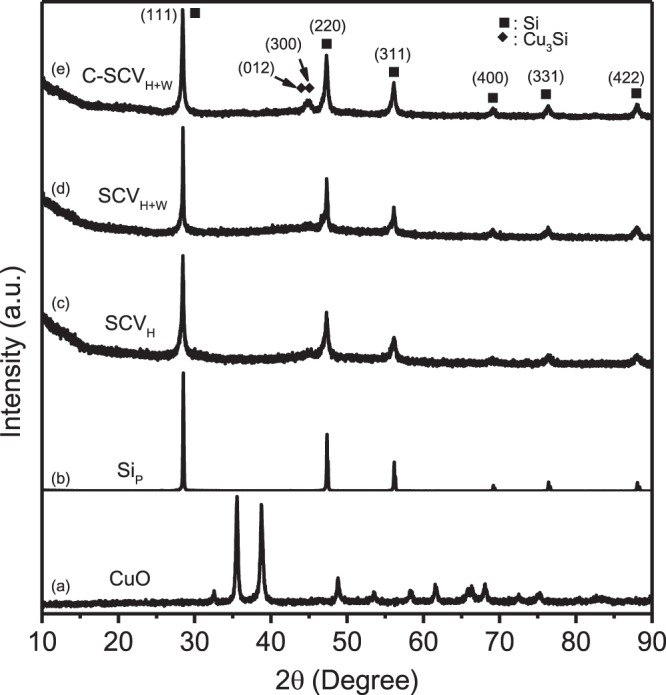
X-ray diffraction patterns of the (**a**) CuO, (**b**) Si_P_ , (**c**) SCV_H_, (**d**) SCV_H+W_ and (**e**) C-SCV_H+W_.

XRD patterns of the CuO, Si_P_, SCV_H_, SCV_H+W_ and C-SCV_H+W_ powders for 2 theta from 10 to 90 degree are shown in Fig. 4. CuO presents peaks (JCPDS card No. 48-1548) at 35.5°, 38.7°, 48.7°, 61.5° and 66.2° (Fig. 4(a)). As well, Si_P_ demonstrates peaks (JCPDS card No. 65-1060), indexing to (111) at 28.4°, (220) at 47.3°, (311) at 56.1° and (422) at 88.0° (Fig. 4(b)). After high energy mechanical milling for mixture of CuO, Si_P_ and VGCF, the micro-sized SCV_H_ shows the broaden peaks similar as Si_P_ without observation of peaks of CuO. CuO in the SCV_H_ composite is suspected to decompose and form amorphous Cu-Si solid solution due to high energy impacting upon HEMM (Fig. 4(c)). Followed by moderate wet milling, submicro-sized SCV_H+W_ (Fig. 4(d)) shows no significant difference from micro-sized SCV_H_ in XRD pattern. After pyrolysis at 800 °C for C-SCV_H+W_, Cu_3_Si peaks (JCPDS card No. 51-0916) indexed to (300) at 45.0° and (012) at 44.6° appear as shown in Fig. 4(e). The average crystallite size of Si calculated by the Scherrer formula from XRD patterns for SCV_H+W_ and C-SCV_H+W_ is 21 nm and 23 nm, respectively. It implies that heat energy enhances crystalline Cu_3_Si formation and slightly increases domain size of Si.

**Figure 5:**
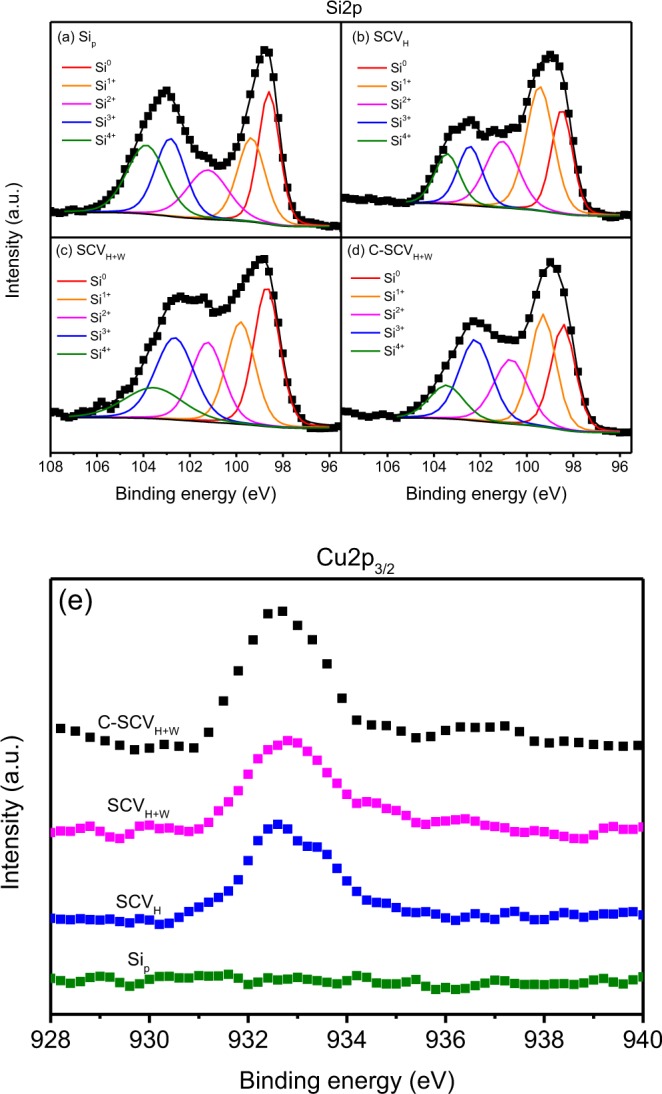
XPS spectra of the Si2p for (**a**) Si_P_ , (**b**) SCV_H_, (**c**) SCV_H+W_, (**d**) C-SCV_H+W_ and (**e**) Cu2p_3/2_ for Si_P_ , SCV_H_, SCV_H+W_ and C-SCV_H+W_, respectively.

The surface composition for Si_P_, SCV_H_, SCV_H+W_ and C-SCV_H+W_ powders is analyzed by X-ray photoelectron spectroscopy (XPS). Figure 5 demonstrates deconvoluted Si2p spectra fitted with five oxidation states with binding energy of 99.2, 100.2, 101.3, 102.1 and 103.4 eV for Si^0^, Si^+^, Si^2+^, Si^3+^ and Si^4+^, respectively^24,25^. Summary of concentrations by integrating the peak intensities for the Si oxidation states of Si2p spectra is shown in Table 1. In all samples as shown in Fig. 5(a–d), the first peak occurring at around 99 eV is assigned to bulk silicon, corresponding to Si^0^. Another peak occurring at around 103 eV is ascribed to SiO_2_, corresponding to Si^4+^. Compared to Si_P_, SCV_H_, SCV_H+W_ and C-SCV_H+W_ show the peak shifting to lower binding energy from 103.0 eV to 102.5 eV. It might be attributed to mechanochemical reaction between CuO and Si upon HEMM resulting in formations of copper silicide and silicon oxide. As compared to Si_P_, Cu2p_3/2_ peak located around 932.8 eV is observed for SCV_H_, SCV_H+W_ and C-SCV_H+W_ (Fig. 5(e)). The binding energy of Cu^2+^ (CuO) located around 934.0 eV shifts to lower binding energy around 932.8 eV, suggesting CuO is reduced^26,27^ upon HEMM.

**Table 1:** Surface concentration measured by XPS deconvoluted by variety of oxidation states for Si2p spectra of Si_P_ , SCV_H_, SCV_H+W_ and C-SCV_H+W_ powders.

Samples	Si^0^ (%)	Si^1+^ (%)	Si^2+^ (%)	Si^3+^ (%)	Si^4+^ (%)
Si_p_	23.3	19.1	16.7	19.3	21.7
SCV_H_	21.1	32.2	21.0	13.8	12.0
SCV_H+W_	25.6	20.7	18.3	22.5	12.9
C-SCV_H+W_	22.4	25.0	19.4	23.2	10.0

Herein, Si/Cu_3_Si-based composite is synthesized by solid-state reaction combining HEMM with heat treatment of carbonization at 800 °C. The sequence is proposed as following reaction (1) and reaction (2). First, CuO is reduced to Cu by Si upon HEMM due to high energy impacting (reaction (1))^28^. Simultaneously, embryo Cu could diffuse into amorphous Si (reaction (2))^29^ in excess nanocrystalline/amorphous Si-rich matrix. Followed by carbonization, heat energy further enhance the crystallinity of Cu_3_Si. Otherwise, silicon oxide seems to exist in shape of amorphous even after heat treatment at 800 °C for C-SCV_H+W_.

1 $$2{\rm{CuO}}+{\rm{Si}}\to 2{\rm{Cu}}+{{\rm{SiO}}}_{2}$$

2 $$3{\rm{Cu}}+{\rm{Si}}\to {{\rm{Cu}}}_{3}{\rm{Si}}$$

**Figure 6:**
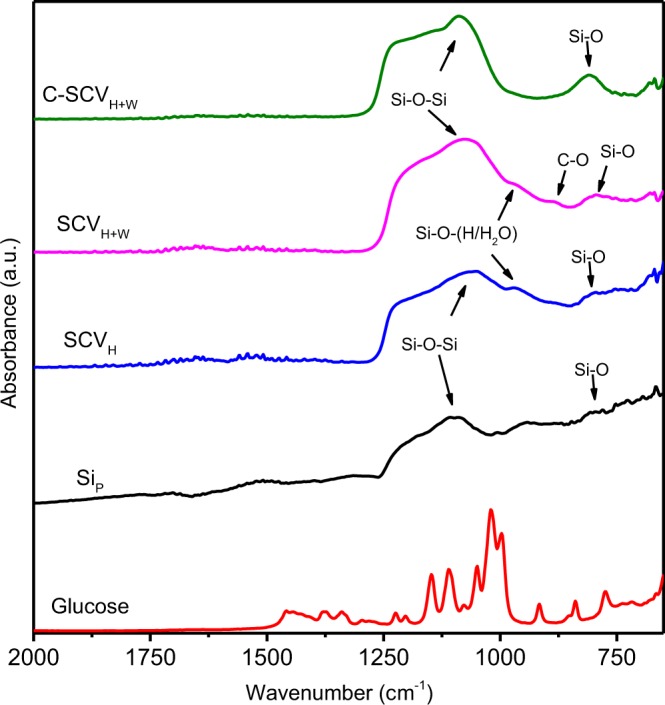
ATR-FTIR spectra for glucose, Si_P_ , SCV_H_, SCV_H+W_ and C-SCV_H+W_, respectively.

ATR-IR spectra of glucose, Si_P_, SCV_H_, SCV_H+W_ and C-SCV_H+W_ present in Fig. 6. The broad band between 1025 and 1257 cm^−1^ can be assigned to Si–O–Si asymmetric stretching and 800 cm^−1^ to Si–O symmetric stretching band, respectively, revealing oxide layer on the surface of these samples. Compared with Si_P_ , SCV_H_ and SCV_H+W_ exhibits peak around 966 cm^−1^, assigning to Si–O–H deformation vibration. It might be ascribed to adsorption of H_2_O from ambient surroundings by highly reactive dangling bonds on Si surface attributed to HEMM^30^. SCV_H+W_ presents the symmetric stretching vibration of C–O bond^31–33^ after wet milling in ethanol, suggesting that ethanol might be modified on the surface of SCV_H+W_. After carbonization for C-SCV_H+W_, Si–O–H and C–O peaks decrease but Si–O peak around 800 cm^−1^ becomes more significant than Si_P_, SCV_H_ and SCV_H+W_.

### Electrochemical behavior

**Figure 7:**
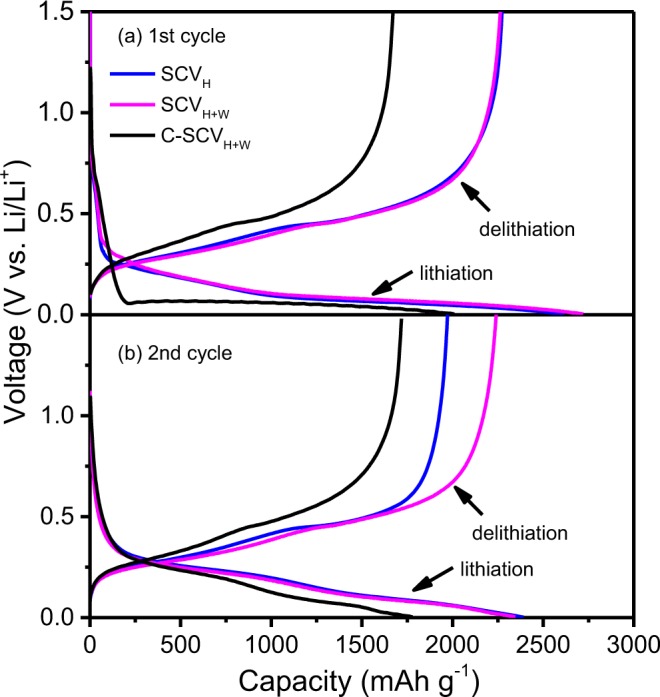
Voltage profiles of SCV_H_, SCV_H+W_ and C-SCV_H+W_ for (**a**) 1^st^ cycle and (**b**) 2^nd^ cycle conducted at current density 200 mA g^−1^ between 0.002 V to 1.5 V.

Voltage vs. capacity for coin cells with negative electrodes of SCV_H_, SCV_H+W_ and C-SCV_H+W_ in the 1^st^ and 2^nd^ lithiation/delithiation cycle are shown in Fig. 7(a) and (b), respectively. In the lithiation/delithiation cycles, current density is 200 mA g^−1^ and voltage is ranged between 0.002 V and 1.5 V at room temperature. In the first cycle, initial specific charge (delithiation) capacities is 2273 mAh g^−1^ for SCV_H_, 2264 mAh g^−1^ for SCV_H+W_ and 1671 mAh g^−1^ for C-SCV_H+W_. Columbic efficiencies of the three negative electrodes in the first cycle are 86.9%, 83.2%, and 83.2%, respectively. C-SCV_H+W_ performs distinguished lithiation from SCV_H_ and SCV_H+W_, existing a steep potential drop from 1.5 to 0.06 V vs. Li/Li^+^ (0 to ~200 mAh g^−1^) in the first cycle. It could be attributed to recovery of crystallite size for Si (i.e. 21 nm and 23 nm for SCV_H+W_ and C-SCV_H+W_, respectively) and residual carbon coating derived from glucose carbonization on C-SCV_H+W_ surface, resulting in various SEI formations.

Supplementary Fig. S3(a) and (b) online present the cyclic voltammetry curves of first and second cycle for SCV_H+W_, C-SCV_H+W_ and Si_H+W_, respectively in a voltage window of 0.005 to 2.0 V at a scan rate of 0.1 mV s^−1^. Here, Si_H+W_^23^ is pure Si processed high mechanical milling and wet milling as the one of SCV_H+W_. Accordingly, onset voltage of first cathodic curves is 0.36 V for SCV_H+W_ and 0.13 V for C-SCV_H+W_, corresponding to observation of steep potential dropping for C-SCV_H+W_ as shown in Fig. 7(a). The different onset voltage in first cycle between SCV_H+W_ and C-SCV_H+W_ might be ascribed to glucose-derived carbon coated on surface of C-SCV_H+W_, resulting in particular SEI formations. After SEI formation, onset voltage of second cathodic curves for C-SCV_H+W_ is started similar with SCV_H+W_ (Fig. S3(b)), consistent with observation in Fig. 7(b). Otherwise, the cathodic peaks around 0.23 V and 0.09 V could be attributed to the alloying reaction upon lithiation between Si and lithium, resulting in phase transformation of Li_x_Si. The sharp anodic peak at around 0.09 V might be ascribed to the lithium stripping current from the lithium accumulation on surface of SCV_H+W_ during cathodic polarization. Two broad anodic peaks (0.29 and 0.47 V) can be attributed to the two-step delithiation of Li_x_Si. In addition, we compare cyclic voltammetry between SCV_H+W_ and Si_H+W_. The examination of the first lithiation scan of cyclic voltammetry (Fig. S3(a)), the onset voltage (0.36 V) and cathodic peaks (0.23 V, 0.09 V) for SCV_H+W_ present slightly shifting to higher voltage than Si_H+W_, onset voltage (0.30 V) and cathodic peaks (0.20 V, 0.05 V). Moreover, the first delithiation scan shows that anodic peaks (0.47 V, 0.29 V) for SCV_H+W_ slightly shift to lower voltage than Si_H+W_, anodic peaks (0.50 V, 0.33 V). This implies that the formation of Cu_3_Si in pure Si could relatively enhances lithiation/delithiation kinetics.

**Figure 8:**
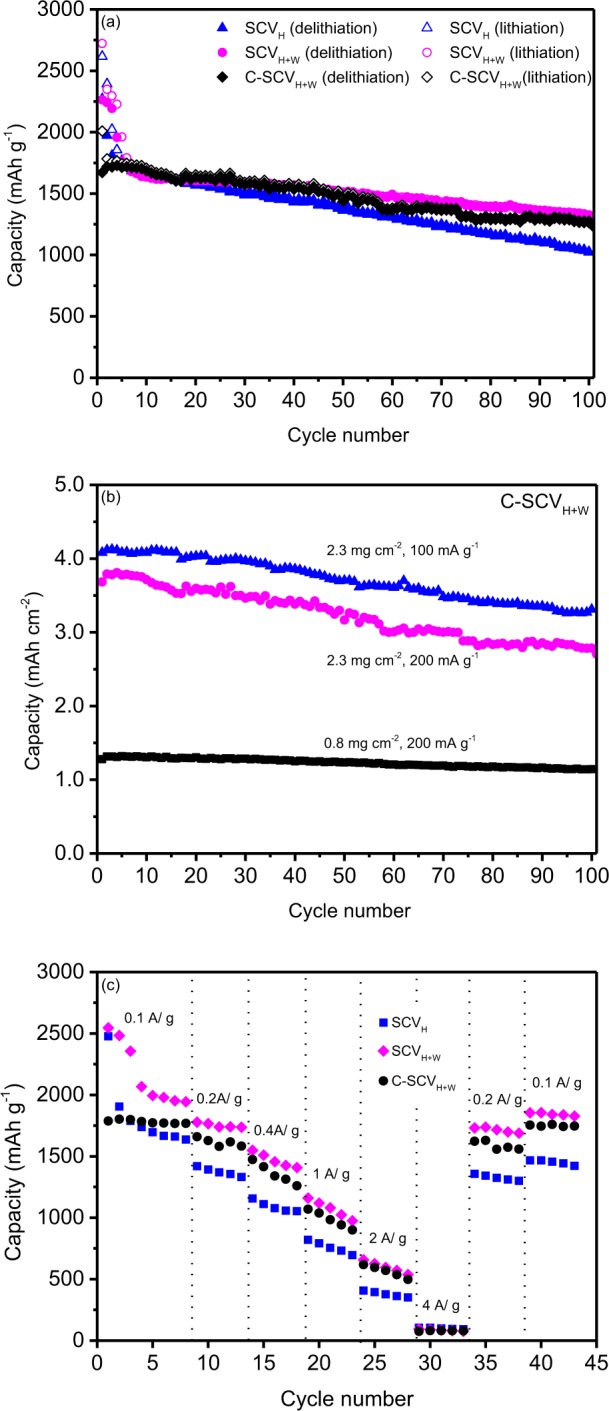
(**a**) Cycling performance of specific discharge (lithiation)/charge (delithiation) capacity for SCV_H_, SCV_H+W_ and C-SCV_H+W_ conducted at current density 200 mA g^−1^ between 0.002 V to 1.5 V. (**b**) Areal charge (delithiation) capacity comparison for C-SCV_H+W_ of varied loading (0.8/2.3 mg cm^−2^) conducted at current density 100 and 200 mA g^−1^ between 0.002 V to 1.5 V. (**c**) Charge (delithiation) capability of SCV_H_, SCV_H+W_ and C-SCV_H+W_ at various charge and discharge rates conducted at current density 0.1, 0.2, 0.4, 1.0, 2.0, 4.0 then 0.2, 0.1 A g^−1^ between 0.002 V to 1.5 V.

Cycling tests of experimental electrodes conducted at 200 mA g^−1^ in a voltage range of 0.002–1.5 V (vs. Li^+^/Li) in specific capacity is shown in Fig. 8(a). Initial capacity and retention capacity after 100 cycles are summarized in Table 2. Compared to SCV_H_ and SCV_H+W_, suppression of fading for C-SCV_H+W_ within initial cycles could be contributed to residual carbon coated on surface as buffer space which leaving sufficient room for relaxing volume expansion between composite particles in lithiation/delithiation processes. Moreover, the construction of nanocrystalline Cu_3_Si distributed in amorphous/nanocrystalline Si matrix is suspected to enhance the conductivity and strength inside the Si/Cu_3_Si-based composite. Thus, the specific charge (delithiation) capacities of SCV_H_, SCV_H+W_ and C-SCV_H+W_ after 100 cycles are 1022 mAh g^−1^, 1317 mAh g^−1^ and 1224 mAh g^−1^, corresponding to retention of 45.0%, 58.2% and 73.2%.

**Table 2:** Electrochemical properties of SCV_H_, SCV_H+W_ and C-SCV_H+W_ conducted at 200 mA g^−1^ between 0.002 V to 1.5 V.

Samples	1st discharge capacity (mAh g^−1^)	1st charge capacity (mAh g^−1^)	1st Columbic efficiency (%)	100th capacity (mAh g^−1^)	Retention (%)
SCV_H_	2617	2273	86.9	1022	45.0
SCV_H+W_	2722	2264	83.2	1317	58.2
C-SCV_H+W_	2009	1671	83.2	1224	73.2

The specific capacity was calculated based on the mass of active materials.

In order to investigate effects of active materials loading and current density on cycling performance for C-SCV_H+W_, varied current density (100/200 mA g^−1^ in a voltage range of 0.002–1.5 V (vs. Li^+^/Li)) and active materials loading (0.8/2.3 mg cm^−2^) are examined in areal capacity variation. Results of initial areal capacity (delithiation) and retained capacity after 100 cycles are summarized in Table 3. Figure 8(b) presents the trend that C-SCV_H+W_ of lower loading (0.8 mg cm^−2^) or lower conducted current density (100 mA g^−1^) performs better relatively retained capacity. However, too low active materials loading means lower areal capacity to practical application in LIBs. To the optimum practice, coin cell made by C-SCV_H+W_ as negative electrode of active materials loading, 2.3 mg cm^−2^ conducted at 100 mA g^−1^ enables an initial charge (delithiation) capacity 1812 mAh g^−1^ (4.08 mAh cm^−2^) with columbic efficiency of 83.7% and 1470 mAh g^−1^ (3.31 mAh cm^−2^) at the end of the 100^th^ cycle. Rate capabilities of SCV_H_, SCV_H+W_ and C-SCV_H+W_ at current density 0.1, 0.2, 0.4, 1.0, 2.0, 4.0 then 0.2, 0.1 A g^−1^ between 0.002 V to 1.5 V are compared in Fig. 8(c). SCV_H+W_ and C-SCV_H+W_ exhibits better rate performance than SCV_H_.

**Table 3:** Effects of active materials loading and current density on areal capacity (delithiation) performance for C-SCV_H+W_ conducted between 0.002 V to 1.5 V.

Loading (mg cm^−2^)	Current density (mAh g^−1^)	1st areal capacity (mAh cm^−2^)	100th areal capacity (mAh cm^−2^)	Retention (%)
0.8	200	1.28	1.14	89.1
2.3	100	4.08	3.31	81.1
2.3	200	3.68	2.69	73.1

In addition, electrodes of blank Cu collector, pristine C-SCV_H+W_ and 100th cycled C-SCV_H+W_ are characterized by XRD in 2θ range from 42.5° to 48.0° as shown in Fig. S4. For pristine C-SCV_H+W_ electrode (Fig. S4(b)), diffraction peak at 47.3° is attributed to the (220) for Si. In addition, diffraction peaks at 44.6°, 45.0° are attributed to the (012) and (300), respectively for Cu_3_Si. After repeatedly lithiation/delithiation reactions, Si crystalline phase transforms into amorphous phase as shown in Fig. S4(c). In contrast, diffraction peaks of (012) and (300) for Cu_3_Si are clearly observed after lithiation/delithiation process.

## Discussion

In this work, the robust method to synthesize Si/Cu_3_Si-based composite as negative electrode materials for lithium ion battery is disclosed. Our results reveal that high energy mechanical milling on Si/CuO mixture induces mechanochemical reaction, resulting in CuO decomposing, amorphizing and alloying with Si. In addition, Si is transformed from well crystalline structure to nanocrystalline/amorphous construction upon high energy impacting. Followed by carbonization, heating energy leads Cu_3_Si to crystallizing and distributing in nanocrystalline/amorphous Si-rich matrix as rigid and conductive enhancer. Furthermore, residual carbon on surface of composite as buffer space alleviates the volume change upon lithiation/delithiation processes. Thus, coin cell made of C-coated Si/Cu_3_Si-based composite (active materials loading, 2.3 mg cm^−2^) as negative electrode conducted at 100 mA g^−1^ enables an initial charge capacity 1812 mAh g^−1^ (4.08 mAh cm^−2^) with columbic efficiency of 83.7% and 1470 mAh g^−1^ (3.31 mAh cm^−2^) at the end of the 100^th^ cycle. Such a performance enables the Si/Cu_3_Si-based composite as potential candidate for negative electrode materials for lithium ion battery.

## Methods

### Materials Preparation

Commercial silicon powder (Si_P_, 99.9%, 10 µm, Fuzhou Hokin Chemical Technology, China), CuO powder (99.9%, 0.7 um, Nissin Chemco, Japan) and vapor grown carbon fiber (VGCF, Showa Denko K.K, Japan) were used as starting materials without further purifying. Si, CuO and VGCF powders were weighted stoichiometrically with weight ratio of 89: 10: 1. The mixture powder was loaded into stainless vial of 500 ml along with stain-less balls of 3 mm in dimension. The ball-to-powder mass ratio was 20:1. The vials were sealed under Ar atmosphere in the glove box. High energy mechanical milling (HEMM) was carried out at room temperature for 9 hours using a planetary miller (PM 400, Retsch, Germany) with a rotation speed of 300 rpm. The milled mixture was denoted as SCV_H_ (micro-sized aggregate). Then, SCV_H_ is introduced into polypropylene jar along with ZrO_2_ balls of 2 mm dimension and ethanol medium. The ball-to-powder mass ratio was 10:1. The wet milling was carried out at room temperature using a rotation mixer at 250 rpm. After wet milling, powder was filtered and dried at 120 °C in vacuum for 12 hours. The wet milled SCV_H_ was denoted as SCV_H+W_ (submicro-sized aggregate). Particle size distribution at 50%, D50 for SCV_H+W_ was controlled around 0.4 μm. Meanwhile, SCV_H+W_ was mixed with glucose (weight ratio 1: 0.4) in ethanol then dried at 120 °C in vacuum for 12 hours. The dried mixture of SCV_H+W_/glucose was placed in an alumina crucible, followed by loading into the alumina tube furnace. The temperature was increased to 800 °C at heating rate 10 °C min^−1^ and held for 1 hour under a mixture gas flow of argon (150 ml min^−1^), hydrogen (20 ml min^−1^). Then, the furnace was nature cooled, while the argon gas (150 ml min^−1^) was continuously flushed through the alumina tube until to room temperature. The carbonization of glucose modified SCV_H+W_ was denoted as C- SCV_H+W_.

### Characterization

The morphology was observed by field emission scanning electron microscope (Carl Zeiss AURIGA CrossBeam (FIB-SEM) Workstation) using an accelerating voltage of 5 kV and high-resolution transmission electron microscope (HRTEM, JEM-2100F, JEOL) with an accelerating voltage of 200 kV. Si composite powders were dispersed in ethanol, and then transferred onto a Formvar carbon coated nickel grid for TEM sample preparation.

The crystal structure was characterized by X-ray diffractometer (D8 ADVANCE Eco) with Cu Kα radiation (λ = 0.15418 nm). Particle size distribution was determined by the laser scattering method using a laser diffraction particle size analyzer (Horiba LA960). Chemical surface composition analysis of the Si powders was conducted using X-ray photoelectron spectroscopy (XPS, PHI 5000 VersaProbe) equipped with an Al Kα X-ray radiation source operating at 1486.6 eV. All spectra were calibrated using C (sp^2^) as a reference binding energy of 284.6 eV. Thermogravimetric analysis (TGA) was carried out using thermogravimetric analyzer (TA Instruments, USA) from 25 °C to 800 °C with a heating rate of 10 °C min^−1^ under air atmosphere. Fourier transform infrared (FTIR) measurement of the Si powders was performed on attenuated total reflectance (ATR) mode using a Cary 660 (Agilent Technologies) FT-IR system equipped with an MCT detector. All spectra were scanned from 650 cm^−1^ to 2000 cm^−1^ with resolution of 2 cm^−1^ for 256 scans.

### Electrochemical Measurements

The slurry was prepared by combining the solid components as 65 wt% of SCV_H_, SCV_H+W_ and C-SCV_H+W_, respectively, 20 wt% of Super P (MMM Carbon, Blegium), 9 wt% of poly(acrylic acid) (PAA, Sigma-Aldrich Co.), 2.5 wt% of carboxymethyl cellulose (CMC, Sigma-Aldrich Co.) and 3.5 wt% of styrene butadiene rubber (SBR, Zeon Co.) in deionized water. Mixing was carried out by a planetary mixer (G-Mixer 400 S, Gold Max Applied Materials Co.) at 500 rpm for 60 min. Electrodes were prepared by casting the slurry onto a sheet of copper foil (Nippon Foil Co.) and dried in the oven at 90 °C for 1 hour. The anodes were stored in a glove box (with oxygen and humidity content maintained below 10 ppm) for more than 24 hours before electrochemical testing. The 2032 coin-type cells were assembled, consisting of a test electrode, lithium foil as a counter electrode and a glass fiber separator (GA-100, Advantec) in an Ar-filled glove box. Electrolyte solution was 1 M LiPF_6_ in a mixture of ethylene carbonate (EC, Ferro Co.), dimethyl carbonate (DMC, Ferro Co.) (1:1 by weight) and additive of 10 wt% fluorinated ethylene carbonate (FEC, Sigma-Aldrich Co.). For the cycle test, the cells were conducted in galvanostatic discharge (lithiation)/charge (delithiation) tests between 0.002 and 1.5 V versus Li^+^/Li using a multi-channel battery testing system (AcuTech Sys-tems BAT-750B). The specific capacity was calculated based on the mass of active materials. Cyclic voltammetry (CV) measurement was carried out using an Autolab electrochemical analyzer (Autolab PGSTAT30, Eco Chemie) with scan rate of 0.1 mV s^−1^ and a potential range of 0.005–2 V (vs. Li^+^/Li). All electrochemical tests were performed at 25 °C.

## Electronic supplementary material

Supporting Information
