# Supplementary material for: Mechanochemical synthesis of Si/Cu3Si-based composite as negative electrode materials for lithium ion battery
Source: Sci Rep. 2018 Aug 23;8:12695. doi: 10.1038/s41598-018-30703-3 (PMC6107536; doi:10.1038/s41598-018-30703-3)
Supplement: Supplementary file 1 — Supporting Information [file 41598_2018_30703_MOESM1_ESM.docx]

Supplementary Information for

**Mechanochemical synthesis of Si/Cu_3_Si-based composite as negative electrode materials for lithium ion battery**

**Shang-Chieh Hou^1^, Tsan-Yao Chen^2^, Yu-Hsien Wu^3^, Hung-Yuan Chen^3^, Xin-Dian Lin^3^, Yu-Qi Chen^3^, Jow-Lay Huang^1,4,5, *^ and Chia-Chin Chang^3,5,**^**

^1^ Department of Materials Science and Engineering, National Cheng Kung University, Tainan 70101, Taiwan

^2^ Department of Engineering and System Science, National Tsing Hua University, Hsinchu 30013, Taiwan

^3^ Department of Greenergy, National University of Tainan, Tainan 70005, Taiwan

^4^ Center for Micro/Nano Science and Technology, National Cheng Kung University, Tainan 70101, Taiwan

^5^ Hierarchical Green-Energy Materials (Hi-GEM) Research Center, National Cheng Kung University, Tainan 70101, Taiwan

Corresponding author:

^*^Jow-Lay Huang E-mail: jlh888@mail.ncku.edu.tw

Telephone number: +886-6-2348188

Fax number: +886-6-2763586

^**^Chia-Chin Chang E-mail: ccchang@mail.nutn.edu.tw

Telephone number: +886-6-2606123 ext 7208

Fax number: +88662602205

**Contents of supporting information**


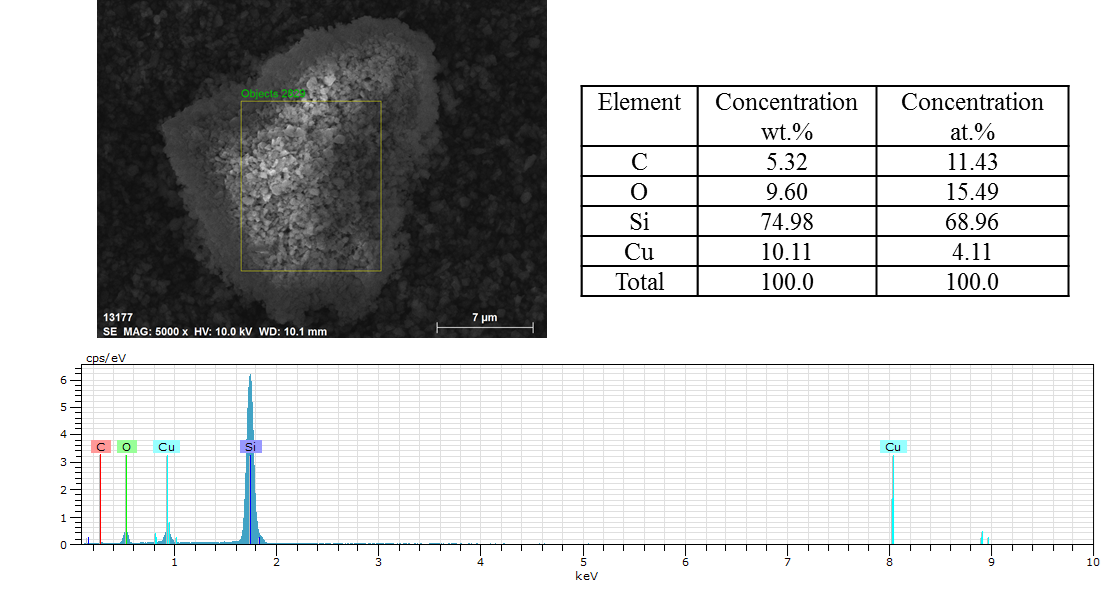


**Figure S1.** Element analysis of SEM-EDS spectrum for C-SCV_H+W_.

**Figure S2.** Thermogravimetric analysis (TGA) profiles for C-SCV_H+W_ sample. The sample was heated to 800 ^o^C at a constant rate of 10 ^o^C/min under air atmosphere. The weight loss is around 4.7 wt% at 550 ^o^C.

**Figure S3.** Cyclic voltammetry profiles of SCV_H+W_, C-SCV_H+W_ and Si_H+W_ for (a) first cycle and (b) second cycle in a voltage window of 0.005–2.0 V at a scan rate of 0.1 mV s^-1^.

**Figure S4.** X-ray diffraction patterns of the electrodes for (a) blank Cu collector, (b) pristine C-SCV_H+W_ and (c) 100^th^ cycled C-SCV_H+W_.
